# Supplementary material for: Target pollen isolation using automated infrared laser-mediated cell disruption
Source: Quant Plant Biol. 2022 Dec 21;3:e30. doi: 10.1017/qpb.2022.24 (PMC10095853; doi:10.1017/qpb.2022.24)
Supplement: Supplementary file 1 [file qpbsup.zip › S2632882822000248sup001.pdf]

Table S1. Time required for disappearance of fluorescein diacetate (FDA) signals in irradiated pollen (se

Laser power at the focal plane (mW)	The time for laser irradiation				
	1 msec	2 msec	5 msec	10 msec	20 msec
24	ND[a]	ND[a]	ND[a]	ND[a]	ND[a]
48	190.0	225.0	155.0	155.0	165.0
96	78.3	91.7	81.7	86.7	76.7
145	70.0	92.5	52.5	42.5	25.0
194	37.5[b]	35.0[b]	32.5[b]	0.0[b]	0.0[b]

[a]ND means that FDA fluorescent signals did not disappear after 5 min of laser irradiation.

[b]The pollen observed to jump and move from the focal plane after irradiation.
